# *Drosophila* parasitoid wasps bears a distinct DNA transposon profile

**DOI:** 10.1186/s13100-018-0127-2

**Published:** 2018-07-07

**Authors:** Alexandre Freitas da Silva, Filipe Zimmer Dezordi, Elgion Lucio Silva Loreto, Gabriel Luz Wallau

**Affiliations:** 1 Pós Graduação em Biociências e Biotecnologia em Saúde, Instituto Aggeu Magalhães (IAM), Recife, Pernambuco Brazil; 2 Departamento de Bioquímica e Biologia Molecular, Universidade Federal de Santa Maria, Santa Maria, Rio Grande do Sul Brazil; 3 Departamento de Entomologia, Instituto Aggeu Magalhães (IAM), Fundação Oswaldo Cruz (FIOCRUZ/PE), Recife, Pernambuco Brazil

**Keywords:** Transposable elements, Repetitive elements, Evolution, Vertical transfer, Active mobilome

## Abstract

**Background:**

The majority of Eukaryotic genomes are composed of a small portion of stable (non-mobile) genes and a large fraction of parasitic mobile elements such as transposable elements and endogenous viruses: the Mobilome. Such important component of many genomes are normally underscored in genomic analysis and detailed characterized mobilomes only exists for model species. In this study, we used a combination of de novo and homology approaches to characterize the Mobilome of two non-model parasitoid wasp species.

**Results:**

The different methodologies employed for TE characterization recovered TEs with different features as TE consensus number and size. Moreover, some TEs were detected only by one or few methodologies. RepeatExplorer and dnaPipeTE estimated a low TE content of 5.86 and 4.57% for Braconidae wasp and 5.22% and 7.42% for *L. boulardi* species, respectively. Both mobilomes are composed by a miscellaneous of ancient and recent elements. Braconidae wasps presented a large diversity of *Maverick/Polintons* Class II TEs while other TE superfamilies were more equally diverse in both species. Phylogenetic analysis of reconstructed elements showed that vertical transfer is the main mode of transmission.

**Conclusion:**

Different methodologies should be used complementarity in order to achieve better mobilome characterization. Both wasps genomes have one of the lower mobilome estimates among all Hymenoptera genomes studied so far and presented a higher proportion of Class II than Class I TEs. The large majority of superfamilies analyzed phylogenetically showed that the elements are being inherited by vertical transfer. Overall, we achieved a deep characterization of the mobilome in two non-model parasitoid wasps improving our understanding of their evolution.

**Electronic supplementary material:**

The online version of this article (10.1186/s13100-018-0127-2) contains supplementary material, which is available to authorized users.

## Background

Transposable elements (TEs) are genetic elements discovered originally in maize (*Zea mays*) by Barbara McClintock [1]. Since then, geneticists have found that they are ubiquitous and can account for a large fraction of some genomes, such as 50 and 85% of primates and maize genomes [2]. Together, TEs and endogenous viruses compose the eukaryotic genome mobilome and such genomic parasites hijack the host molecular machinery for their own replication [3]. New copies of those parasites may generate deleterious mutations to the host genome but most of the observed insertions are probably slightly deleterious or neutral to the organism [4]. However, accumulating evidence shows that the TEs can also generate genetic variability that can be co-opted for a new host functions [5–7].

A robust characterization of the mobilome in several species is essential to evaluate evolutionary interplay between between genomic parasites and host species [8]. Well-characterized mobilomes are restricted to model organisms while it remains largely unexplored in non-model species. However, genome-wide non-model organisms studies are revealing different genomic parasites/host dynamics which differ substantially from the most studied ones. It highlights that our current view of genome and mobilome evolution focusing on few well-studied species is likely biased.

A large number of bioinformatic softwares are available for genome-wide TEs characterization, but most of them require a complete or draft assembly genome as input [9–11]. However, depending on the fragmentary nature of draft assemblies TE characterization can be largely compromised [12]. In order to characterize the mobilome in the absence of a genome assembly new approaches were developed [13] and further improved allowing the characterization of the mobilome directly from a large number of small sequenced reads [10, 14]. A growing number of studies are characterizing the mobiome of non-model organisms using these approaches [15], such as the grasshopper (*Gomphocerus sibiricus*) [16], killifish (*Austrolebias charrua*) [17], the Asian Tiger Mosquito (*Aedes albopictus*) [18] and the repetitive landscape of different *Musaceae* species [19].

Insect genomes have a huge variability in TE diversity and content but overall a direct relation of TE content and genome size can be seen with a higher abundance of Class I over Class II TEs [20, 21]. However, when analyzing the main eukaryotic taxa (plants, fungi and some other specific animal taxa) no clear pattern emerges showing that TE content and diversity only correlates with the evolutionary history of the studied species [22, 23]. It highlights the need for better and robust mobilome characterization in organisms from a more diverse set of taxa in order to test the associations between TE content/diversity and genome size [23].

Parasitoid wasps are insects from the Hymenoptera order particularly known due to their specific relationship with several arthropod species. Females of parasitoid wasp species inject venom and deposit eggs on or inside of their hosts [24]. The venom is composed of several molecules along with viral-like particles (VLPs), which are responsible for the inhibition of the host’s immunological system. VLPs also can act as a vector of DNA fragments between wasps and their hosts [25–27]. Wasp genomes are underrepresented in genomic surveys and so far only four genomes are available: three closely related *Nasonia* genomes (Pteromalidae family) and the *Fopius arisanus* genome (Braconidae family) [28]. However, only *Nasonia vitripennis* genome has a reported mobilome showing one of the highest high TE content (28.8%) among Hymenopteran genomes [24].

This study aimed to describe and compare the mobilome of two *Drosophila* parasitoid wasps: *Leptopilina boulardi* (Figitidae family) and a wasp from the *Aphidius* genus (Braconidae family) and evaluate their evolutionary history. We demonstrated that these genomes have a large diversity of TE superfamilies with ancient and recent TEs. Moreover, phylogenetic reconstruction of each superfamily showed that the majority of elements identified were transmitted through vertical transfer.

## Methods

### Samples and DNA sequencing

Wasp specimens were sampled at Santa Maria City, latitude 34.95303 and longitude − 120.43572 parasitizing *Drosophila* flies - *Leptopilina boulardi* parasites several *Drosophila* species while Braconidae species is a restricted parasite of *Drosophila* species from the flavopilosa group - a highly specialized species group that uses flowers as unique breeding sites [29–31]. It is important to emphasize that we tried to identify the Braconidae wasp at the lowest taxonomic level possible, but after contacting specialized taxonomists it was not possible to reach species identification. Therefore, we will call this species from now on as braconid or Braconidae wasp although it is closely related to the *Aphidius* genus in a previous COI analysis [29–31]). Genomic DNA was prepared with TruSeq DNA HT Sample Prep Kit (Illumina) according to the manufacturer’s instructions and sequenced in a Solexa-Illumina HiSeq 2000 New Generation Sequencing (NGS) device using a single-end approach of read length of 100 bp [29].

### Transposable elements characterization

Characterization and evolutionary study of TEs from wasps were performed following the pipeline in (Additional file 1).

We have used two complementary approaches to characterize the mobilome from raw Illumina reads: I) Raw reads were used as input for RepeatExplorer (RE) analysis pipeline with default parameters. Wasp datasets were independently analyzed. Raw reads clustering were performed using an all-to-all similarity comparison which builds a graph relative to each group of a repetitive element [14]. RepeatExplorer annotated the reads of each assembled cluster using RepeatMasker (http://www.repeatmasker.org) [15] against the Repbase database [32]. Following, we sought to characterize the top clusters (clusters that represent more than 0.01% of the reads used) having the majority of the reads with BLAST hit to a known Repbase TE. Resulting top clusters contigs were then reassembled using CAP3 [33] with the following parameters (−a 20 -b 20 -c 12 -d 200 -e 30 -f 20 -g 6 -m 2 -n 5 -p 80 -r 1 -s 900 -t 300 -u 3 -v 2 -o 40) as used by others [17, 34] (Additional file 1). II) dnaPipeTE [10] were run with two Trinity iteractions and variable amount of reads to evaluate its performance and find the best parameter set. Final parameters are as follow: -sample_size 14,000,000 (the maximum number of reads allowed using two trinity iteractions, considering that we have around 28Mi reads for each wasp species), −sample_number 2 and -RM_t 0.5. Those two approaches were used to estimate the proportion of each TE class and superfamilies in the two genomes (Additional file 1).

Additionally, we performed one de novo characterization using RepeatScout 1.0.5 (RS) [35] using the original assembly obtained from Ortiz et al. 2015. Finally, we clustered three TE libraries generated by these programs plus the Ortiz et al. 2015 TE library, characterized by BLASTn against Repbase, using CD-HIT-EST 4.6 [36] with parameters (−c 0.8 -G 0 -aS 0.8 -g 1 -M 50000 -T 8 -n 5) to generate the final TE dataset for each wasp (Additional file 1).

### Evolutionary analysis

In order to reconstruct the evolutionary history of each TE superfamily reliably one need to obtain the largest coding region of each consensus assembled. Hence, we performed open read frame (ORFs) searches only in the RepeatExplorer TE dataset (largest consensus obtained) using getorf (> 100aa) implemented in EMBOSS package [37]. CD-SEARCH was used to identify TE conserved domains [38] against the CDD database (CDD v3.16–50,369 PSSMs). All analysis after RepeatExplorer clusterization was performed independently in the respective standalone softwares (CAP3 and getorf). After that we recovered homologous sequences using three different strategies. I - a homology search using protein sequences with known TE domains against Repbase using CENSOR [39] with default parameters. Sequences with the best scores were retrieved for each search. II - BLASTp searches against non-redundant protein sequence (nr) NCBI database with default parameters. Up to 50 protein sequences with the best scores were retained after removing specie-specific redundancies. III - Literature review relative to each superfamily studied and curated TE sequences were retrieved. The Repbase, Literature and BLASTp retrieved sequences were analyzed through getorf [40] to recover their potential coding regions (Additional file 1).

Alignment of each superfamily was performed using protein sequences with more than 100 amino acids using MAFFT [41] and edited manually to remove highly variable regions. Such an approach was taken instead of automatic software due to the high variability in the ORFs size and amino acid composition in TE protein sequences. For instance, even loosening GBlocks parameters almost all alignments sites were removed in the final alignment of some superfamilies (data not shown). Phylogenetic trees were built through PhyML [42] and protein substitution models were evaluated through Smart Model Selection implemented in the same PhyML server (http://www.atgc-montpellier.fr/phyml/). Trees were visualized and colored using FigTree 1.4.3 [43] and iTOL web server [44].

### Sequence similarity

A sequence similarity analysis, one per superfamily, was performed with the MEGA 7 software [45], to evaluate possible horizontal transposon transfer events and characterize monophyletic groups in the phylogenetic trees. Additionally, we also performed Kimura two parameters (K2P) distance analysis between each read and the consensus generated for each contig in order to estimate the relative age of the family inside each genome. Only contigs assembled with RE plus CAP3 and used in the phylogenetic analysis were included due to their higher average size and better recovery of complete or almost complete TE coding regions. We first obtained the ACE assembly file where all reads were aligned to the multiple contigs obtained from each cluster, then we extracted a multiple sequence alignment of all reads mapped against the contigs that presented the ORF with the TE conserved domain. Then such MSA was given as input to MEGA 7 to estimate K2P distance.

## Results

### TEs characterization by different softwares

#### TEs detection

Overall TEs detected by the different softwares differed in number varying from 74 to 10,267 in braconid wasp and 105 to 12,796 in *L. boulardi* and average size varying from 113 to 601 in the braconid wasp and from 105 to 672 in *L. boulardi* genome (Additional file 2).

Graph-based clustering analysis in RepeatExplorer yielded 661.605 and 787.848 clusters from each wasp respectively*.* Considering only top clusters, 313 and 516 top clusters were retained from braconid and *L. boulardi* wasps. Eighty-one top clusters from Braconidae wasp and 54 from *L. boulardi* showed homology with TEs sequences from Repbase, representing respectively 5.86% and 5.22% of wasp genomes (Additional file 3), with Class II TEs being the most abundant in both species. A total of 18 and 15 TEs superfamilies were found in Braconidae and *L. boulardi* wasps, respectively (Additional file 3).

**Fig. 1:**
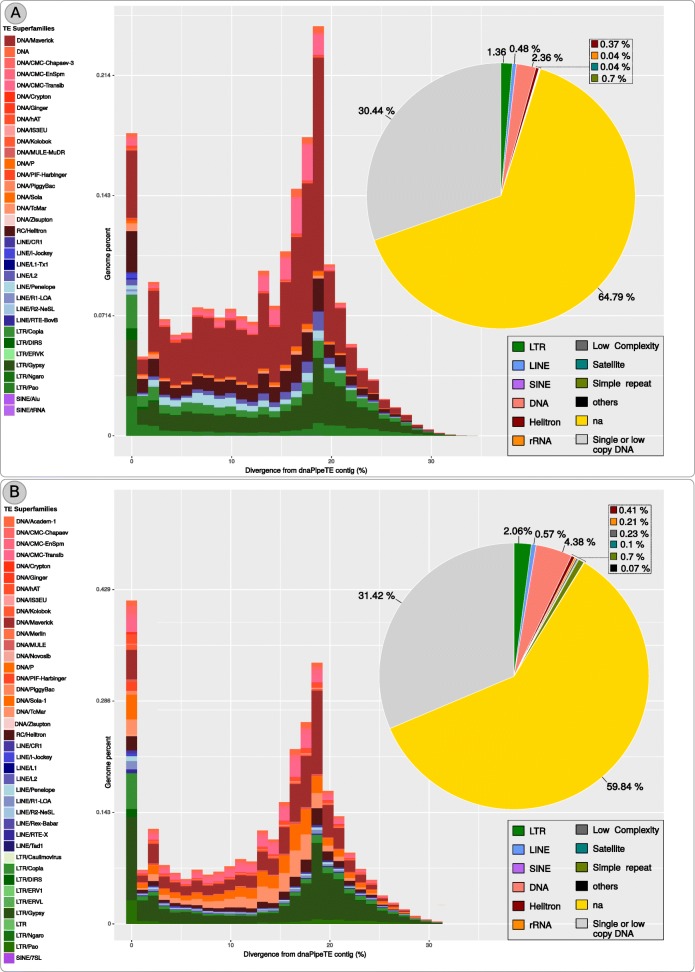
Charts representing the proportion of each repetitive genomic component of the two wasp species. **a** braconid wasp and **b** *L. boulardi*. Pie Chart with the overall proportion of reads by each repetitive type and landscape with proportion (y-axis) and relative age analysis (x-axis - K2P) by each TE superfamily. Right legends correspond to major repetitive types found depicted in the pie chart graph (na - non annotated) and left legends correspond to TE superfamilies depicted in the landscape graph

TEs assembly performed with dnaPipeTE yielded 10,267 and 12,796 annotated elements from Braconidae and *L. boulardi* species representing 4.57 and 7.42% of each genome respectively (Fig. 1 a and b). Class II TEs were the most abundant in both wasps genomes with 2.73 and 4.79% of the estimated TE content while Class I correspond to 1.84 and 2.63% of Braconidae and *L. boulardi* genomes respectively (Fig. 1 a and b). A total of 29 and 36 TEs superfamilies were found in Braconidae and *L. boulardi* genomes (Additional file 3).

In the Braconidae wasp all 18 superfamilies detected in the RE top clusters were also detected by dnaPipeTE while in *L. boulardi* 14 out of 15 TE superfamilies detected in the RE top clusters were also recovered by dnaPipeTE (*LOA* superfamily was detected only through RE analysis) (Additional file 3). In addition, 11 and 21 superfamilies were only detected by dnaPipeTE in the Braconidae and *L. boulardi* genomes respectively. Such differences were expected based on the analysis of only top cluster in the RE analysis.

#### Final consensus reconstructed by each strategy

**Fig. 2:**
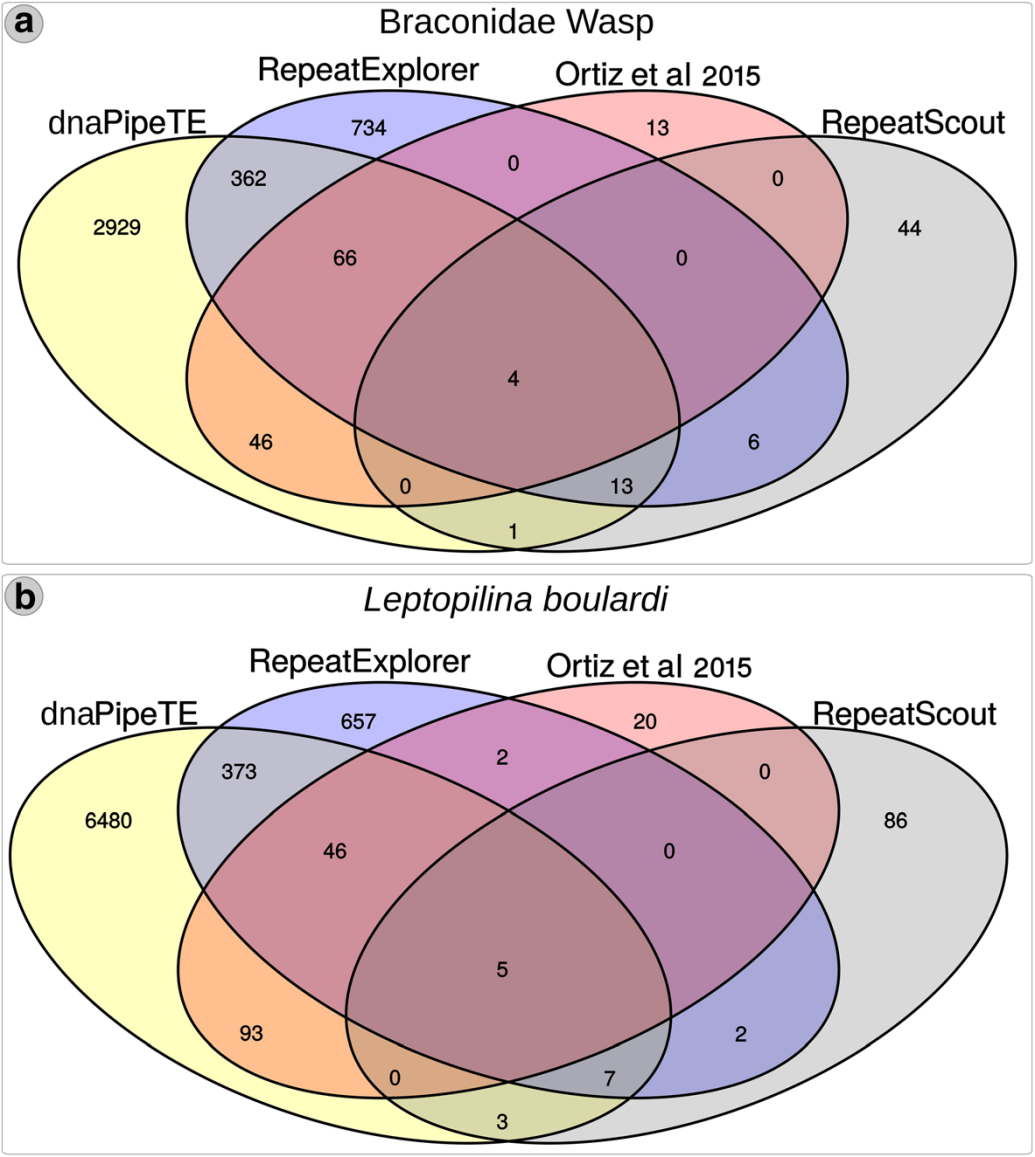
Venn diagram with cd-hit-est clusterization of final TE contigs from each of the four analysis performed for TE characterization. **a** braconid wasp and **b** *L. boulardi*

In order to generate a final TE library combining all methodologies used to characterize the wasps mobilome (RepeatExplorer, dnaPipeTE, RepeatScout and the data provided by Ortiz et al. 2015) we clustered the resulting contigs following the 80–80 rule [46]. We could detect that only a minor subset of 4 and 5 sequences in Braconidae and *L. boulardi* wasps were characterized by all 4 methodologies and a large overposition occurred between RE and dnaPipeTE with 362 and 373 sequences recovered by these two methods (Fig. 2a and b). The two methods that recovered the largest amount of unique elements was dnaPipeTE (2929 and 6480) and RepeatExplorer (734 and 657) for both wasps (Fig. 2a and b), but RepeatScout (44 and 86) and Ortiz et al. 2015 (13 and 20) library also presented unique sequences.

### Intragenomic dynamics

**Fig. 3:**
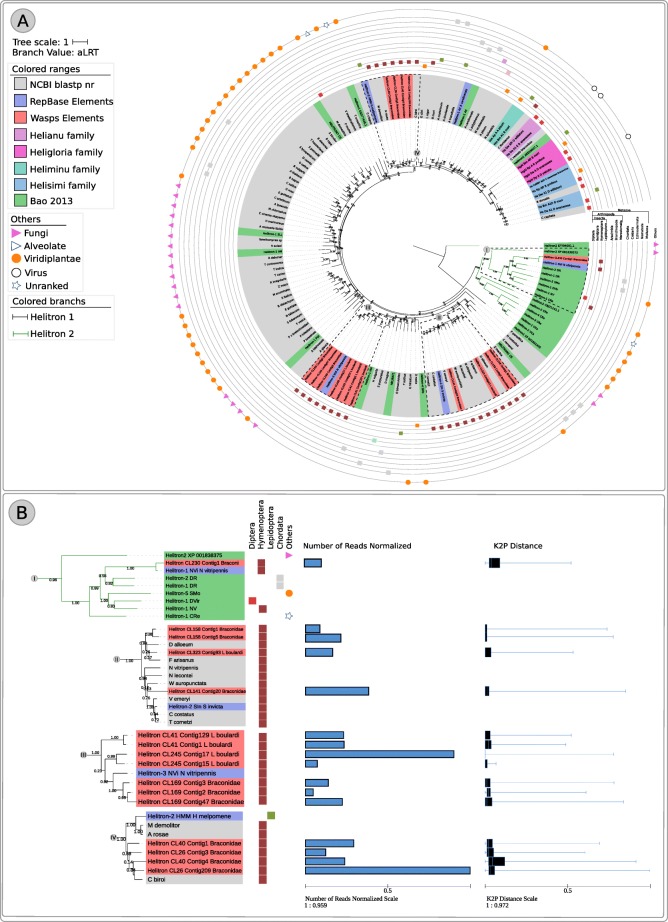
Maximum likelihood *Helitron* phylogenetic reconstruction using Helicase protein sequences from Repbase, literature and NCBI search along with *Helitron* sequences from the wasps studied. Tip colors represent sequences recovered from RepBase - blue and NCBI database - grey. Moreover, orange, light green, purple and pink are the *Helitron* families described by Thomas et al. 2010 and black and green branch colors denotes *Helitron* 1 and 2 described by Bao and Jurka 2013 respectively. The number over nodes are aLRT estimates of node support. **a** Full phylogeny including all sequences sampled. Dashed squares represent the clades zoomed in part B. **b** Zoom on four clades encompassing wasp *Helitrons*. Blue bars are total reads used to assemble each contig normalized by the contig size and bluish boxplot is the average, and Q1/Q3 quartile and bars represent the maximum and minimum values. Online data about *Helitron* phylogeny is available on: https://itol.embl.de/tree/20013326113391523965160#

**Fig. 4:**
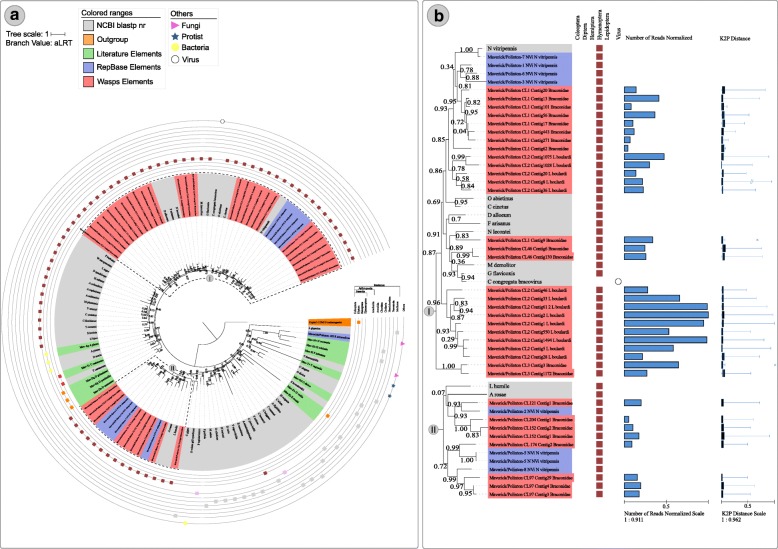
Maximum likelihood *Maverick/Polintons* superfamily phylogenetic reconstruction using integrase protein sequences from Repbase, literature and NCBI search along with *Maverick/Polintons* sequences from the wasps studied. Tip colors represent sequences recovered from RepBase - blue, from the literature - green and NCBI database - black. The number over the nodes are aLRT node support. **a** Full phylogenetic tree and **b** Zoomed tree clades highlighted in the dashed squares from part A. Blue bars are total reads used to assemble each contig normalized by the contig size and bluish boxplot is the average and Q1/Q3 quartile and bars represent the maximum and minimum values. Online data about *Maverick/Polintons* phylogeny is available at: https://itol.embl.de/tree/2001332610271781508755345#

**Fig. 5:**
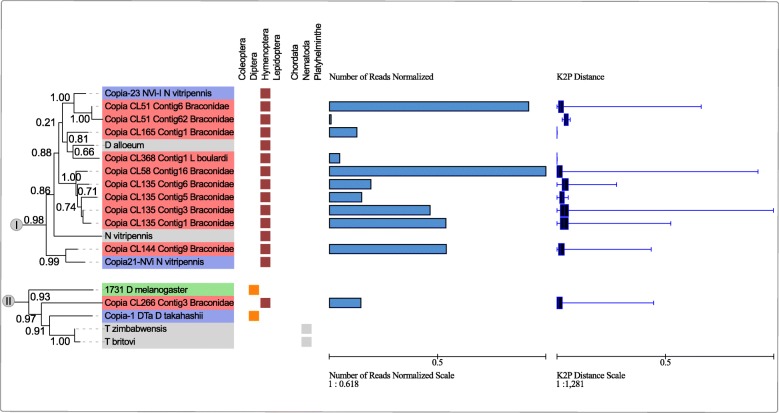
Maximum likelihood of *Copia* superfamily phylogenetic reconstruction using full protein sequences from Repbase, literature and NCBI search along with *Copia* sequences from the wasps studied. Tip colors represent sequences recovered from RepBase - blue, from the literature - green and NCBI database - black. The number over the nodes are node support aLRT estimates. I and II are zoomed clades from full phylogeny which can be found in Additional file 9. Online data about *Copia* phylogeny is available at: https://itol.embl.de/tree/2001332610130171508862213

**Fig. 6:**
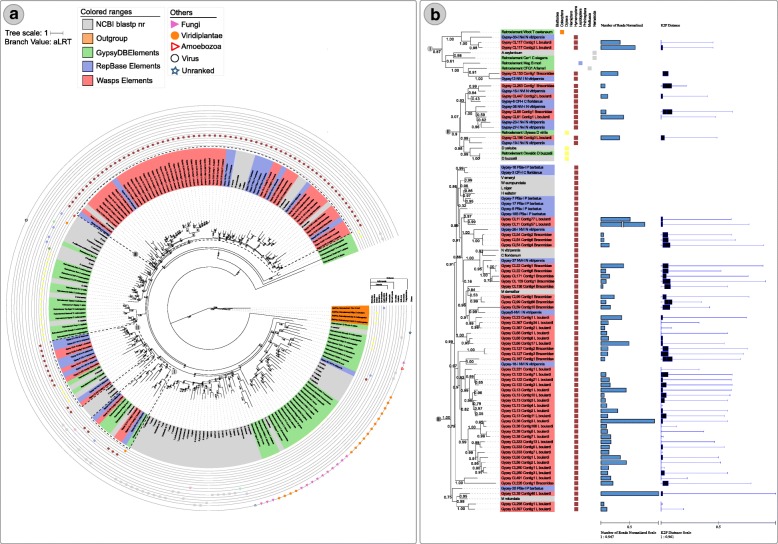
Maximum likelihood of *Gypsy* superfamily phylogenetic reconstruction using full protein sequences from Repbase, literature and NCBI search along with *Gypsy* sequences from the wasps studied. Tip colors represent sequences recovered from RepBase - blue, from the literature - green and NCBI database - black. The number over nodes are aLRT estimates of node support. **a** Full phylogenetic tree and **b** Zoomed tree clades highlighted in the dashed square from part A. Blue bars are total reads used to assemble each contig normalized by the contig size and bluish boxplot is the average, and Q1/Q3 quartile and bars represent the maximum and minimum values. Online data about *Gypsy* phylogeny is available at: https://itol.embl.de/tree/177183205251262901509016275

Overall both wasps have a mobilome characterized by a mixture of ancient and young elements with representatives of all major superfamilies in the dnaPipeTE analysis (Fig. 1 a and b). Additionally, we performed abundance and relative dating analysis within species on the RE reconstructed elements used in the phylogenetic analysis. A different relative abundance can be observed among elements reconstructed from the same genome. Such data can be seen in section B of Figs. 3, 4, 5 and 6. For instance, in clade II of Fig. 3b we can see that the element *Helitron*_CL245_Contig17_L_boulardi has the highest proportion of normalized reads in comparison to other closely related *Helitron* elements from *L. boulardi* genome. Another striking example can be seen in clade III in the *Gypsy* superfamily tree (Fig. 6b) where some elements presented a much higher relative abundance compared with other elements of the same species. Although we could see some patterns with a higher proportion of reads and lower K2P element age we could not detect any strong negative correlation (Pearson correlation between 0.3 and − 0.3) inside each superfamily (data not shown). Overall, RE and dnaPipeTE relative age estimate were congruent showing that there is a young fraction of the mobilome in both wasps which is related to recent activity of these elements.

### Deeper TE superfamily characterization on RE reconstructed consensus

Only contigs from RE analysis showing superfamily-specific proteins domains (Additional file 4) were used for phylogenetic reconstruction since they presented largest contig sizes compared with the other strategies used in this study (Additional file 2). After manual alignment quality check, final alignments have smaller protein amino acid sequences ranging from around 62% of its original size (in *Gypsy* superfamily) to only 20% (in *L2* superfamily) (Additional file 5). Such large reduction in the *Gypsy* superfamily is probably due to the large diversity of proteins included in our analysis since this superfamily has the highest number of sequences used in the alignment and phylogenetic reconstruction (196) compared with other superfamilies. We were able to reconstruct the evolutionary history of seven superfamilies including elements from both *L. boulardi* and the braconid wasp (subsections below): Class II - DNA transposon - *Helitron*, *Maverick/Polintons* and Class I Retrotransposons - *Copia*, *Gypsy*, *L2*, *BEL* and *Penelope* (Additional file 6). Moreover, four phylogenetic trees were reconstructed only with *L. boulardi* sequences: Class II - DNA transposons - *Chapaev* and Class I - Retrotransposons - *Loa*, *I* and *R1* (Additional file 6). All sequences used in each phylogenetic analysis are presented in Additional file 7.

### The *Helitron* superfamily

Known as rolling-circle elements, these TEs were first described in the genomes of plants (*Arabidopsis thaliana* and *Oryza sativa*) and in the nematode *Caenorhabditis elegans* [47]. Further studies identified homologous elements in several other genomes, such as protists, arthropods and mammals [48]. *Helitrons* are characterized by a RepHel region (Rep from Replication initiator and Hel from Helicase domain) and other protein domains such as Cysteine-Protease, Apurinic-Endonuclase, Zinc-Finger and Protein Replication A [48].

We used 16 contigs characterized with RE (11 from braconid wasp and 5 from *L. boulardi*) to reconstruct their evolutionary history*.* Those elements clustered into four divergent lineages (Fig. 3), two of those composed of sequences of TEs from both waps (Fig. 3 BII-BIII) and the other two with braconid sequence only (Fig. BI-BIV). All 16 contigs clustered outside of previous defined *Helitron* families [49] (orange, green, pink and purple in Fig. 3a) but 15 of those clustered inside of *Helitron* 1 while only 1 braconid sequences clustered inside *Helitron* 2 clade defined by Bao and Jurka 2013 (Fig. 3a - black and green branches denotes *Helitron* 1 and 2 respectively): BI presents the single braconid *Helitron* clustered within the *Helitron* 2 cluster showing close phylogenetic relationship with the *Helitron*-1 element from *N. vitripennis* available at Repbase (Fig. 3b). BII clade grouped *Helitrons* from *L. boulardi* and braconid wasps with other wasp species such as *N. vitripennis*, *Diachasma alloeum* (Braconidae), *Fopius arisanus* (Braconidae) and four ant species (*Wasmannia auropunctata, Vollenhovia emeryi, Cyphomyrmex costatus, Trachymyrmex cornetzi*) (Fig. 3b). BIII clade grouped three braconid and four *L. boulardi* elements with *Helitron*-3 element from *N. vitripennis* described in Repbase (Fig. 3b). BIV grouped Braconidae *Helitrons* sequences with an ant, *Cerapachys biroi* *Helitron* sequence found in NCBI (Fig. 3 a and b, Additional file 8). Overall, *Helitrons* from wasp species clustered with each other and were closely related to ants, being congruent with host species phylogeny [50].

### The *Maverick/Polintons* superfamily

TEs of this superfamily have been found in protist, fungi and animal genomes and are characterized as one of the largest and structurally complex TEs described to date (10-15 kb) which codify four proteins probably involved in their transposition mechanism: DNA-Polymerase-B, Retroviral-Integrase, Cysteine-Protease and ATPase [51, 52].

Here we identified 36 RE contigs with Retroviral-Integrase protein domains (22 from braconid wasp and 14 from *L. boulardi*) which were used to reconstruct their evolutionary history (Fig. 4). Wasps *Maverick/Polintons* sequences clustered into two major clades (I and II in Fig. 4 a and b). Clade I presented several contigs of both wasps and several other wasp sequences from databases. Highly supported subclades show a basal clade with only two braconid sequences, a second clade with only *L. boulardi Maverick/Polintons* sequences, a third clade with a single braconid wasp element clustered with *Neodiprion lecontei* sequence and a clade with two braconid wasp sequences, a fourth clade with five *L. boulardi* elements and eight braconid wasp elements clustering with *N. vitripennis* elements (Fig. 4b). While clade II has two clear subclades with braconid wasp *Maverick/Polintons* and *N. vitripennis*, *Linepithema humile and Athalia rosae* sequences (Fig. 4b). Overall, *Maverick/Polintons* evolution followed vertical transmission, clustering wasp sequences characterized here with other wasp and Formicidae sequences available.

### The *Copia* superfamily

TEs of this superfamily were first identified in the *Drosophila melanogaster* genome [53] and subsequently in plant genomes [54], but several studies showed that these elements are widely distributed among eukaryotic taxa. Elements from this superfamily codify a single ORF with two domains (GAG and POL - GAG is associated with structural proteins responsible for TE genetic material packing in viral like particles (VLPs - similar to retroviruses), POL genes which are responsible for TE replication and translocation [55]) and long terminal repeats in both 5′ and 3′ element extremities [56].

Eleven RE contigs presenting a reverse-transcriptase domain were analyzed phylogenetically (ten from braconid wasp and 1 from *L. boulardi*) (full phylogeny can be found in Additional file 9 and clades bearing TEs from wasps in Fig. 5). *Copia* elements clustered into two divergent lineages, one encompassing 9 braconid wasp elements and a single *L. boulardi* element (Fig. 5 - Clade I). Clade I elements clustered with three *N. vitripennis* (Pteromalidae) elements (*Copia*-23 and *Copia*-21) and one element from the parasitoid wasp *D. alloeum* (Braconidae) (Fig. 5 - Clade I) showing an early branch of this clade composed of a *Copia* element from *Trichogramma pretiosum,* another parasitoid wasp, but from Trichogrammatidae family. Clade II clustered a single braconid wasp element with two *Drosophila* and two nematodes species from the *Trichinella* genus with a high node support (0.91–1, Fig. 5). Such elements presented a distance ranging from 1.22 to 1.59 amino acid changes per site (Additional file 10- red shaded cells).

### The *Gypsy* superfamily

TEs of this superfamily are abundant in plant and animal genomes [57]. They are characterized by two ORFs, GAG and POL, similar to the *Copia* superfamily, but with different protein domains positions [46].

We identified a total of fifty-six RE contigs with the reverse-transcriptase domain, 18 from the braconid wasp and 38 from *L. boulardi*. The *Gypsy* superfamily is the most diverse superfamily found among those parasitic wasps (Fig. 6). Three large clades encompassing wasp *Gypsy* elements can be seen (Fig. 6a and b - Clade I, II and III). Overall, *Gypsy* elements from braconid wasp formed clusters with *N. vitripennis* and *L. boulardi* elements (Fig. 5a and b - Clade I and II). However, some clades were found composed by ant and bees sequences closely related to wasps *Gypsy* elements (Fig. 6b). For instance, *Gypsy*_CL11_Contig77_L_boulardi and *Gypsy*_CL11_Contig57_L_boulardi elements clustered with *Gypsy*-18B_PBa-I_P_barbatus, an ant species *Pogonomyrmex barbatus* with high branch support (0.97) (Fig. 6b - Clade III), and presented an amino acid distance of 0.75 and 0.76 amino acid changes per site. Another example in the same clade is *Gypsy*_CL39_Contig1_L_boulardi which grouped with a bee, *Megachile rotundata*, an element with high branch support (0.96) and an amino acid distance of 0.49 amino acid substitution per site (Fig. 6b - Clade III).

Other interesting branching pattern emerging from the *Gypsy* tree were two clearly defined clusters with only *L. boulardi* elements which suggests that those elements were amplified and diversified successfully in this genome (Fig. 5b - Clade I).

## Discussion

The extraordinary rate in which new genomes are being sequenced allows researchers to have a better view of genome evolution in several different taxa and test the consistency of major patterns driving genome expansion and diversification. However, the mobilome, a dynamic and large fraction of many genomes, is disregarded mainly due to its inherent complexity associated with a long-lasting view that it is not important for understanding the genome evolutionary dynamics. Although some efforts have been made to better characterize the mobilome, we only have a precise characterization for model organisms. Here we described the mobilome of two non-model organisms, a Braconidae wasp (probably a new species from the *Aphidius* genus) and *L. boulardi* wasp species showing that they have a diverse mobilome and a mixture of ancient and young elements. Moreover, TE content and superfamily diversity of DNA transposons differs substantially between them and with other previous studied wasp species.

TE content in eukaryotic genomes varies greatly with some species apparently free of such parasites up to genomes composed of 60 to 80% [2]. In insect the TE content ranges from < 1% in *Belgica antarctica* to 60% in *Locusta migratoria* and *Aedes albopictus* genomes [21]. Species from the Hymenoptera order also have a large variation in TE content as the bee *Apis mellifera* 7.57% [58]*,* and the ant species *Cardiocondyla obscurior* 7.18% [59]*,* and the *Camponotus floridanus* 15.62% [60], *Harpegnathos saltator* 27.53% ant species [60]. Wasps mobilome characterization only exists for three *Nasonia* genomes having a TE content of around 25.8% of their genomes [24] although a recent publication reported a lower estimate: 20% [59]. Braconidae and *L. boulardi* wasps studied here showed the smallest TE contents (considering both RE and dnaPipeTE results - braconid - 5.86/4.57 and *L. boulardi* - 5.22/7.42) compared other Hymenopteran and *Nasonia* genomes*.* However, we need to keep in mind that mobilome comparison among studies is a hard task mainly due to different genome quality and approaches used for TE detection and annotation (see discussion below and references - [23, 61]) and that the genomes studied here represent only a tiny subset of wasp species diversity.

Known insect genomes usually have a higher proportion of Class I than Class II TEs [21]. However, reported Hymenopteran genomes have a higher proportion of Class II (six ants genomes) than Class I (2 genomes including *N. vitripennis*), while *Apis mellifera* showed an almost equal amount of these classes [58, 59]. Braconidae and *L. boulardi* wasps presented a higher proportion of Class II than Class I TEs (Fig. 1). Such differences can be partially explained by the large amount of *Maverick/Polintons* superfamily in both wasp species, being the most abundant superfamily of braconid wasp genome in both RE and dnaPipeTE analysis, and the third and second more abundant in *L. boulardi* genome in RE and dnaPipeTE results (Additional file 3). Other Hymenopterans genomes, such as *Atta cephalotes*, *H. saltator* and *Acromyrmex echinatior* ant species, have a low *Maverick/Polintons* genomic proportion ranging from 0.509, 0.543 and 0.408% compared with the total Class II TE content which was estimated around 7, 7.5 and 8% respectively. While *N. vitripennis* presented a Class II TE content of 7.8% and a *Maverick/Polintons* content of 1.452% [59]. Taking the data presented above, we estimate that the contribution of *Maverick/Polintons* to the total Class II TEs content presented higher values in *N. vitripennis* and Braconidae wasps studied here (18.61 and 32.01%), two species from sister families Braconidae and Pteromalidae, than in the three ant species and *L. boulardi* species where such contribution varied between 5.1 in *A. echinator* up to 12.40% in *L. boulardi* (7.24% in *H. saltator* and 7.27% in *A. cephalotes*). This data suggests that *Maverick/Polintons* superfamily is an important component of the Pteromalidae and Braconidae wasps genomes that probably expanded in the ancestral of these two families around 213 MYA (http://www.timetree.org/). *Maverick/Polintons* seems to contribute to Class II TE in *L. boulardi* as well, besides a high abundance of *Sola*, *Tc1-mariner*, *Helitron* and *Transib* superfamilies is also observed (Additional file 3).

The mobilome of those two wasp species was characterized before using a homology-based approach obtaining a total of 20 superfamilies [29]. In this study, we used a combination of two de novo approaches based on TE reconstruction from raw reads (RE and dnaPipeTE) and one de novo approach that characterize TEs from assembled genomes (RS) in combination with the original TEs described by Ortiz et al. 2015 to generate a final TE dataset for the studies wasps. We were able to identify several new TEs superfamilies and a large overpositon of TEs recovered could be observed among the methodologies employed (Fig. 2a and b). However, each strategy recovered TE contigs with specific features. Given the non-model species studied here and the increase in TE superfamilies and TE consensus size detected by the de novo approach directly from raw reads, such strategy seems to be more appropriate to characterize mobilomes of non-model organisms. Anyhow, those approaches should be used complementary to each other in order to have a more complete view of the mobilome.

The identification of a large number of contigs allowed the extraction of potential coding regions and identification of several TE specific domains which allowed us to get further insight into their evolution (Additional file 4). We were able to reconstruct seven superfamily-level phylogenetic trees, representing the most abundant and diverse superfamilies found by RE. In general, TE superfamilies from braconid wasp studied here are more diversified, that is, have a larger number of contigs (which represent TE families) than *L. boulardi* TE superfamilies with the exception of the *Gypsy* superfamily. Clustering patterns showed that elements from both wasps usually grouped closely with elements characterized in *N. vitripennis,* other wasps, or ant and bee species, all from the Hymenoptera order. Moreover, braconid wasp elements are more related to *N. vitripennis* elements than to *L. boulardi* elements which reflects the phylogenetic relationship of the host species: Braconidae and Pteromalidae (*N. vitripennis*) wasp families are more phylogenetically related than Figitidae family (*L. boulardi*) [29] which is in agreement with the results presented before regarding the diversity and abundance of *Maverick/Polinton* on *N. vitripennis* and braconid wasp. Overall, branching patterns suggests that the majority of elements from the two wasps are evolving through vertical transmission. However, some phylogenetic clusters encompass closely related TEs from host species that split several million years ago can be seen in the *Gypsy* phylogenetic tree: I - *L. boulardi* sequences clustered with *Megachile rotundata,* a bee species, presenting around 70% of similarity at the protein level. These two species diverged from each other around 203 Mya (http://www.timetree.org/). Therefore, at least for *Gypsy* superfamily our data suggests one ancient horizontal transfer event between wasp and bees species although further evidences are needed in order to better evaluate such event.

Another interesting point is that even knowing that *Drosophila* genomes have one of the best-characterized mobilomes [62], we could not find any traces of potential horizontal transfer events between wasps and flies, which supports our previous findings that *Drosophila* parasitoid wasps, and *Drosophila* hosts do not exchange TEs through HT, and that VLPs injected by these wasps are not an important TE vector [29]. This is in contrast with several horizontal transfer events mediated by Lepidoptera parasitoid wasps and their VLPs [25–27, 63]. These contradictory findings suggest that each vector-parasite relationship has different characteristics that either allow or act as a barrier for HT between species and highlights the importance of taking into account non-model organisms when extrapolating results on the evolution of TEs.

Based on our broad analysis including several protein sequences from different databases, we were able to reconstruct comprehensive superfamily phylogenetic trees which brought new information about the evolution of several TEs superfamilies. *Helitron* superfamily tree obtained in this work is the first tree including such extensive *Helitron* diversity. Most *Helitron* studies focused in phylogenetic trees at the nucleotide level which hindered analysis of more distant homologous sequences as well as confident reconstruction of deep ancestral nodes with confidence [49, 64]. There is only one study reporting deep branch nodes of *Helitron* superfamily but that was analyzed with only 29 protein sequences [65]. Our comprehensive tree recovered the two major monophyletic *Helitrons* clades described by Bao and Jurka 2015 showing that parasitoid wasps sequences mostly belong to the *Helitron* 1 clade and only one sequence characterized in this study clustered in the *Helitron* 2 clade. Regarding *Maverick/Polintons*, the last published and more comprehensive analysis used all sequences from this superfamily available at Repbase (56 elements) to reconstructs their evolutionary history [66]. Here we used 88 elements gathered from the literature [67] and available at NCBI along with 36 elements reconstructed from braconid and *L. boulardi* genomes. We obtained the same two major clades with similar topologies recovered by Haapa-Paananem et al. 2014, but our large sampling allowed us to show that ant *Maverick/Polintons* are the sister group of wasp *Maverick/Polintons* and deep understand the diversity of *Maverick/Polintons* from several species of wasps including the one focused on this study.

Regarding the abundance of elements and their intragenomic dynamics we could detect that some elements probably have a higher copy number which is related to a more successful amplification in the host genome. Moreover, we detected that the wasps mobilomes studied here have an ancient mobilome, which is in agreement with the vertical inheritance signal supported by the phylogenetic analysis, but with a similar amount of young TE families which is likely related with recent activity of these elements.

## Conclusion

This study characterized two non-model wasp mobilomes shedding new light on the evolution of those elements and hosts. We detected several TE superfamilies not described before for those species, showed that *Maverick/Polintons* compose an abundant genomic component in Pteromalidae and Braconidae wasps, revisited some TE superfamily phylogeny showing that most of the wasps TEs are evolving vertically and evidenced that the two wasps mobilomes investigated here have a miscellaneous of ancient and young elements which likely contribute to the intragenomic dynamics of such understudied taxa [68–70].

## Additional files

Additional file 1: Workflow of softwares used in this study. A) pipelines and softwares used to complementary characterize the mobilome of both wasp species and derivate a final TE dataset. B) Evolutionary analysis performed only with RepeatExplorer contigs per superfamily. (PDF 31 kb)

Additional file 2: Features of TE contigs recovered by different approaches. (DOCX 8 kb)

Additional file 3: Summary of superfamilies abundance per wasp species analyzed. (DOCX 13 kb)

Additional file 4: Contigs description and TE conserved domains found. (XLSX 38 kb)

Additional file 5: Protein length before and after manual alignment edition. (XLSX 46 kb)

Additional file 6: Species-specific superfamily trees reconstructed by maximum likelihood. The number on branches denote the aLRT branch support. (PDF 4864 kb)

Additional file 7: Final TE contigs after redundancy removal with cd-hit-est characterized in this study. (FASTA 7362 kb)

Additional file 8: *Helitron* phylogenetic tree with complete coding regions of RepBase elements. (PDF 59 kb)

Additional file 9: *Copia* superfamily tree reconstructed by maximum likelihood. Numbers on branches denote the aLRT branch support. (PDF 641 kb)

Additional file 10: Amino acid distance between TEs proteins used in the phylogenetic reconstruction. (XLSX 914 kb)

## Abbreviation

NCBI: National Center for Biotechnology Information
STDEV: standard deviation
TE: transposable element
VLP: viral-like particle
