# Supplementary material for: Drosophila parasitoid wasps bears a distinct DNA transposon profile
Source: Mob DNA. 2018 Jul 7;9:23. doi: 10.1186/s13100-018-0127-2 (PMC6035795; doi:10.1186/s13100-018-0127-2)
Supplement: Supplementary file 1 — Workflow of softwares used in this study. A) pipelines and softwares used to complementary characterize the mobilome of both wasp species and derivate a final TE dataset. B) Evolutionary analysis performed only with RepeatExplorer contigs per superfamily. (PDF 31 kb) [file 13100_2018_127_MOESM1_ESM.pdf]

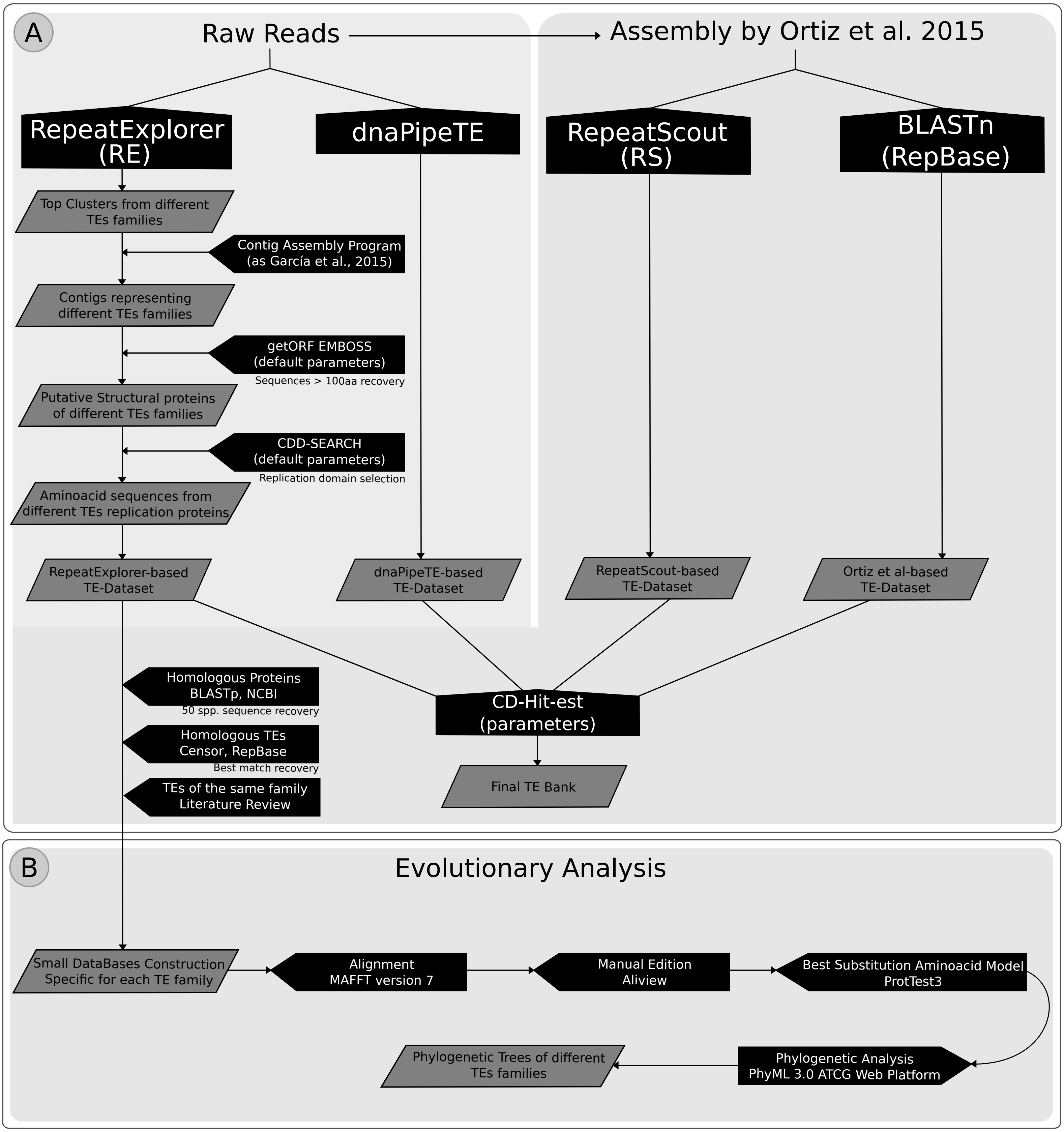

Supplementary Material 1.1 - Pipeline built for this work. **A** - Different strategies for TEs identification. **B** - Evolutionary history reconstruction of different families. Web platforms was next: RepeatExplorer: http://www.repeatexplorer;org/ - CDDSearch: https://www.ncbi.nim.nih.gob/Structure/cdd/wrpsb.cgi - getORF: http://emboss.bioinformatics.nl/cgi-bin/emboss/ - BLASTp: https://blast.ncbi.nlm.nih.gov/Blast;cgi?PAGE=Proteins - Censor, Repbase: http://www.girinst.org/censor/ - MAFFT:https://mafft.cbrc.jp/alignment/server/ - PhyML 3.0: http://www.atgc-montpellie.fr/phyml/
