# Supplementary material for: Drosophila parasitoid wasps bears a distinct DNA transposon profile
Source: Mob DNA. 2018 Jul 7;9:23. doi: 10.1186/s13100-018-0127-2 (PMC6035795; doi:10.1186/s13100-018-0127-2)
Supplement: Supplementary file 2 — Features of TE contigs recovered by different approaches. (DOCX 8 kb) [file 13100_2018_127_MOESM2_ESM.docx]

**Braconidae**

| **Method** | **Number of TE sequences** | **Average of sequences length** **(bp)** | **STdev of sequences length** | **Largest contig length (bp)** | **Smallest contig length (****bp)** |
| --- | --- | --- | --- | --- | --- |
| dnaPipeTE | 10.267 | 410.397 | 387.699 | 5.725 | 201 |
| RepeatExplorer | 1.974 | 601.451 | 907.957 | 10.644 | 100 |
| RepeatScout | 74 | 176.797 | 75.858 | 473 | 100 |
| Ortiz *et al*. 2015 | 475 | 113.787 | 37.925 | 390 | 80 |

***L. boulardi***

| **Method** | **Number of TE sequences** | **Average of sequences length** | **STdev of sequences length** | **Largest contig length (bp)** | **Smallest contig length (bp)** |
| --- | --- | --- | --- | --- | --- |
| dnaPipeTE | 12.796 | 476.899 | 492.505 | 6.984 | 201 |
| RepeatExplorer | 1.447 | 672.229 | 1001.230 | 10.970 | 109 |
| RepeatScout | 105 | 154.866 | 65.663 | 559 | 100 |
| Ortiz *et al.* 2015 | 380 | 105.457 | 35.762 | 516 | 80 |
