# Supplementary material for: Drosophila parasitoid wasps bears a distinct DNA transposon profile
Source: Mob DNA. 2018 Jul 7;9:23. doi: 10.1186/s13100-018-0127-2 (PMC6035795; doi:10.1186/s13100-018-0127-2)
Supplement: Supplementary file 3 — Summary of superfamilies abundance per wasp species analyzed. (DOCX 13 kb) [file 13100_2018_127_MOESM3_ESM.docx]

| **TE type** | ***L. boulardi* TEs superfamilies *** | **Number of Reads RE**** | **% of the genome**  **RE**** | **Number of Repeat Masker hits **** | **dnaPipeTE Reads** | **% of the genome**  **dnaPipeTE** | **Contigs used in Phylogenetic analysis ***** |
| --- | --- | --- | --- | --- | --- | --- | --- |
| **I - DNA Transposons** | *Sola* | 78238 | 1,256 | 23692 | 43172 | 0.308 |  |
|  | *Academ* |  |  |  | 569 | 0.004 |  |
|  | *Maverick/Polintons* | 63237 | 1,019 | 19504 | 83597 | 0.597 | 14 |
|  | *Helitron* | 16285 | 0.262 | 2715 | 24788 | 0.177 | 5 |
|  | *Chapaev* | 11054 | 0,179 | 1111 | 7627 | 0.054 |  |
|  | *EnSpm* |  |  |  | 12319 | 0.088 |  |
|  | *Crypton* |  |  |  | 692 | 0.005 |  |
|  | *hAT* | 6251 | 0.101 | 1787 | 9558 | 0.068 |  |
|  | *Ginger* |  |  |  | 893 | 0.006 |  |
|  | *PiggyBac* | 1456 | 0,024 | 58 | 1341 | 0.010 |  |
|  | *Transib* | 1009 | 0,016 | 158 | 22124 | 0.158 |  |
|  | *IS3EU* |  |  |  | 1686 | 0.012 |  |
|  | *Kolobok* |  |  |  | 5854 | 0.042 |  |
|  | *Merlin* |  |  |  | 105 | 0.001 |  |
|  | *MULE* |  |  |  | 2491 | 0.018 |  |
|  | *Novosib* |  |  |  | 304 | 0.002 |  |
|  | *P* |  |  |  | 78 | 0.001 |  |
|  | *PIF-Harbinger* |  |  |  | 4432 | 0.032 |  |
|  | *Tc1-mariner* |  |  |  | 38719 | 0.277 |  |
|  | *Zator* |  |  |  | 972 | 0.007 |  |
|  | *Zisupton* |  |  |  | 277 | 0.002 |  |
| **II - Non LTR retrotransposons** | *R1* | 13027 | 0,210 | 981 | 8041 | 0.057 |  |
|  | *R2* |  |  |  | 120 | 0.001 |  |
|  | *RTE* |  |  |  | 3224 | 0.023 |  |
|  | *CR1* |  |  |  | 3590 | 0.026 |  |
|  | *L1* |  |  |  | 345 | 0.002 |  |
|  | *L2* | 4412 | 0,071 | 1235 | 792 | 0.006 | 2 |
|  | *Penelope* | 1795 | 0,029 |  | 4740 | 0.034 | 2 |
|  | *Tad1* |  |  |  | 75 | 0.001 |  |
|  | *LOA* | 3056 | 0,049 | 137 |  |  |  |
|  | *I* | 2859 | 0,046 | 69 | 812 | 0.006 |  |
| **II - LTR retrotransposons** | *Gypsy* | 119216 | 1,921 | 16845 | 100445 | 0.717 | 38 |
|  | *BEL* | 3183 | 0,051 | 371 | 13239 | 0.095 | 3 |
|  | *DIRS* |  |  |  | 3992 | 0.029 |  |
|  | *Ngaro* |  |  |  | 116 | 0.001 |  |
|  | *Copia* | 1345 | 0,022 | 162 | 20498 | 0.146 | 1 |
| **Total** |  | 324628 | 5.227 | 68825 | 429794 | 3.070 | 65 |
|  | **Braconidae TEs superfamilies *** |  |  |  |  |  |  |
| **I - DNA Transposons** | Maverick/Polintons | 171209 | 2,260 | 32660 | 122312 | 0.874 | 23 |
|  | *Academ* |  |  |  | 107 | 0.001 |  |
|  | *Kolobok* |  |  |  | 1124 | 0.008 |  |
|  | *MULE* |  |  |  | 286 | 0.002 |  |
|  | *P* |  |  |  | 73 | 0.001 |  |
|  | *PIF-Harbinger* |  |  |  | 1023 | 0.007 |  |
|  | *Helitron* | 30338 | 0,400 | 2630 | 32531 | 0.232 | 11 |
|  | *EnSpm* | 29250 | 0,385 | 482 | 1530 | 0.011 |  |
|  | *Chapaev* | 7226 | 0,096 | 637 | 2620 | 0.019 |  |
|  | *Crypton* | 4481 | 0,060 | 147 | 430 | 0.003 |  |
|  | *Transib* | 23459 | 0,031 | 990 | 15602 | 0.111 |  |
|  | *Tc1-mariner* | 1726 | 0,023 | 106 | 3757 | 0.027 |  |
|  | *Zator* |  |  |  | 138 | 0.001 |  |
|  | *hAT* | 779 | 0,010 | 1 | 1951 | 0.014 |  |
|  | *Sola* | 7442 | 0.097 | 406 | 3360 | 0.024 |  |
| **II - Non LTR retrotransposons** | *Penelope* | 22240 | 0,294 | 1295 | 9506 | 0.068 | 1 |
|  | *R1* |  |  |  | 412 | 0.003 |  |
|  | *R2* |  |  |  | 1761 | 0.013 |  |
|  | *L1* |  |  |  | 156 | 0.001 |  |
|  | *L2* | 11532 | 0,152 | 1267 | 9426 | 0.067 | 9 |
|  | *SINE* | 1614 | 0,021 | 46 | 72 | 0.001 |  |
|  | *CR1* |  |  |  | 283 | 0.002 |  |
|  | *I* |  |  |  | 926 | 0.007 |  |
| **II - LTR retrotransposons** | *Gypsy* | 65718 | 0,867 | 5922 | 49755 | 0.355 | 18 |
|  | *Copia* | 22738 | 0,301 | 3329 | 21402 | 0.153 | 10 |
|  | *Ngaro* | 22336 | 0,295 | 1 | 86 | 0.001 |  |
|  | *BEL* | 12983 | 0,172 | 1207 | 15846 | 0.113 | 4 |
|  | *DIRS* | 5809 | 0,077 | 441 | 2658 | 0.019 |  |
|  | *ERV1* | 1151 | 0,015 | 1 | 15 | 0.000 |  |
| **Total** |  | 444001 | 5.861 | 51692 | 304014 | 2.172 | 76 |

* Black TE superfamilies identified with both RepeatExplorer and dnaPipeTE. Green superfamilies are the ones identified only by dnaPipeTE.

** RE - RepeatExplorer - only top cluster analysed.

*** Only coding regions that presented a potential amino acid chain > 100 aa were used in the phylogenetic reconstruction.
