# Supplementary material for: Drosophila parasitoid wasps bears a distinct DNA transposon profile
Source: Mob DNA. 2018 Jul 7;9:23. doi: 10.1186/s13100-018-0127-2 (PMC6035795; doi:10.1186/s13100-018-0127-2)
Supplement: Supplementary file 6 — Species-specific superfamily trees reconstructed by maximum likelihood. The number on branches denote the aLRT branch support. (PDF 4864 kb) [file 13100_2018_127_MOESM6_ESM.pdf]

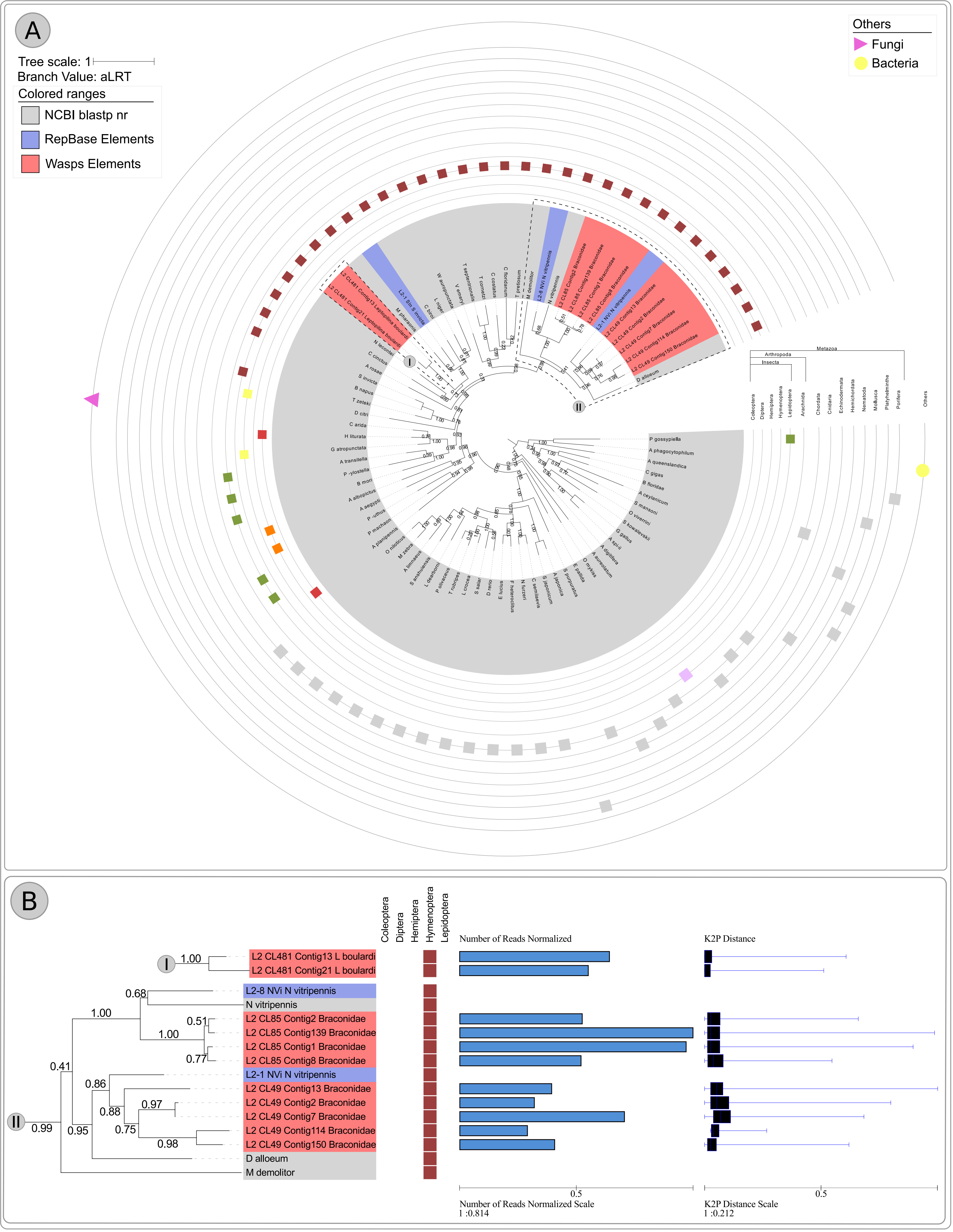

Figure 1S - Maximum likelihood of L2 superfamily phylogenetic reconstruction using full protein sequences from Repbase and NCBI search along with L2 sequence from both wasps. Tip colors represent sequences recovered from RepBase - blue and NCBI database - black. Triangles over nodes are proportional to the node support accessed by aLRT estimates. A) Full phylogenetic tree and B) Zoomed one clade highlighted in the outermost bars from part A. Online data about L2 phylogeny is available on: https://itol.embl.de/tree/2001321431101691510857774

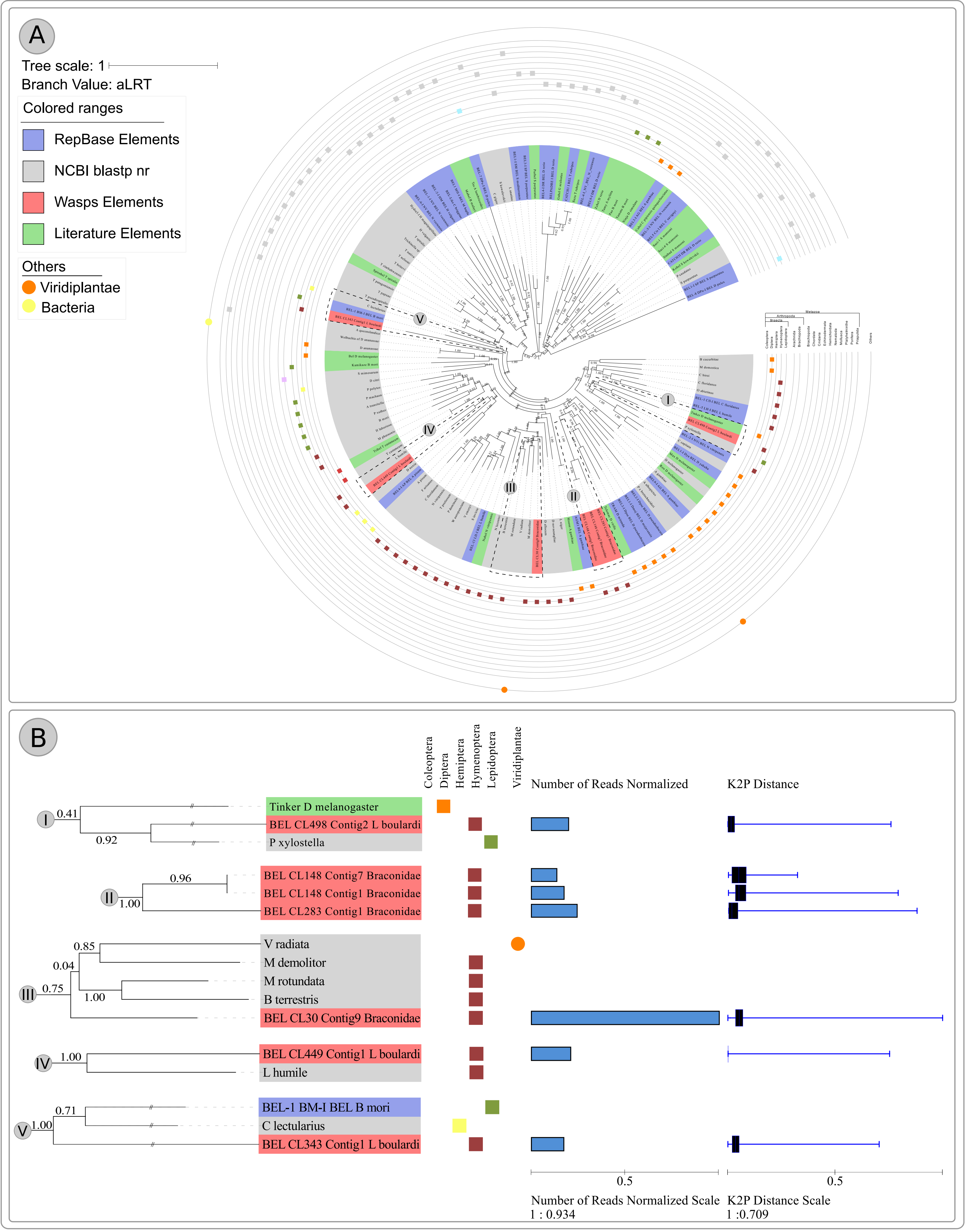

Figure 2S - Maximum likelihood of Bel superfamily phylogenetic reconstruction using full protein sequences from Repbase, literature and NCBI search along with Bel sequence from both wasps. Tip colors represent sequences recovered from RepBase - blue, from the literature - green and NCBI database - black. Triangles over nodes are proportional to the node support accessed by aLRT estimates. A) Full phylogenetic tree and B) Zoomed one clade highlighted in the outermost bars from part A. Online data about Bel phylogeny is available on: https://itol.embl.de/tree/200133261027121507571775

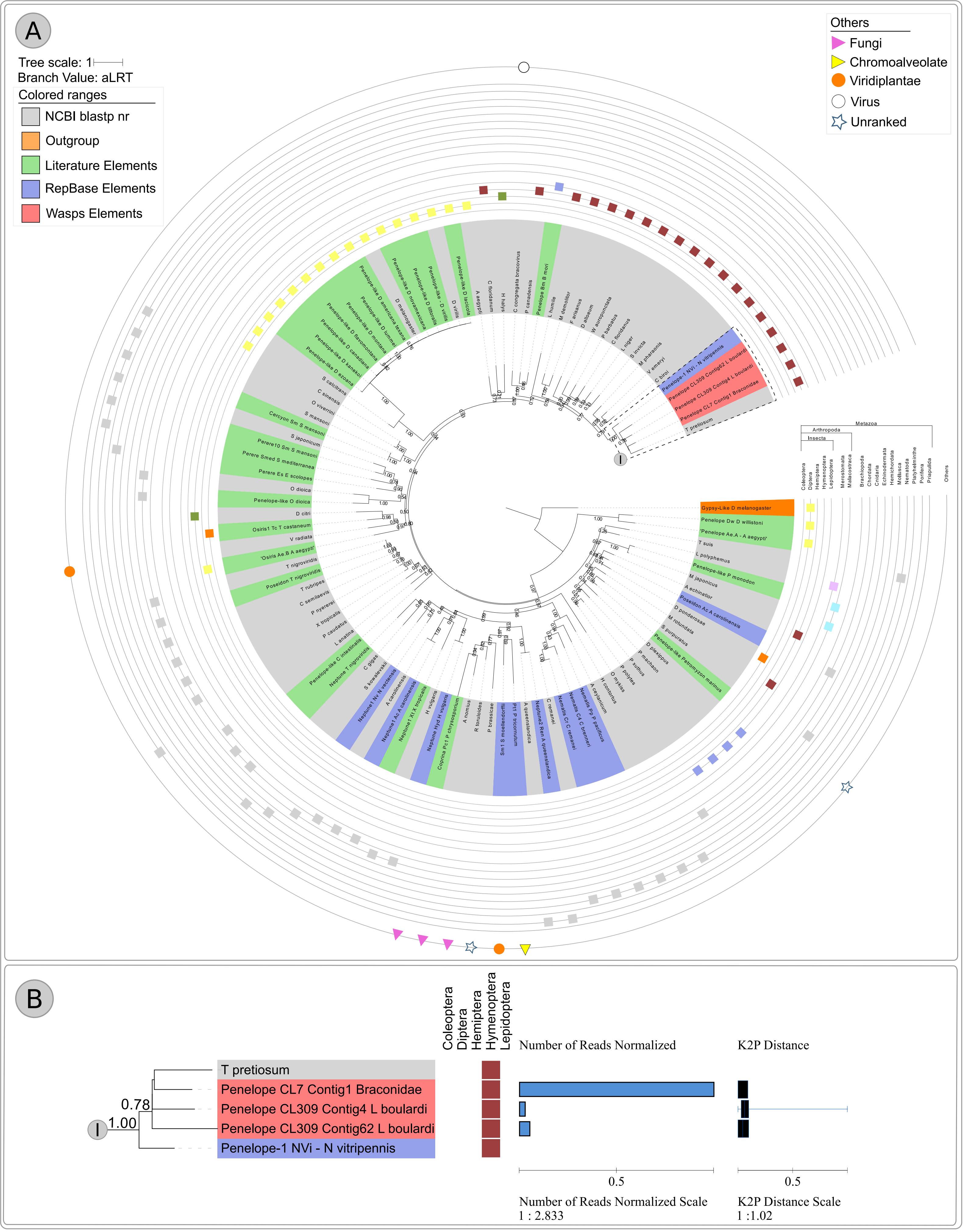

Figure 3S - Maximum likelihood of Penelope superfamily phylogenetic reconstruction using full protein sequences from Repbase, literature and NCBI search along with Penelope sequence from both wasps. Tip colors represent sequences recovered from RepBase - blue, from the literature - green and NCBI database - black. Triangles over nodes are proportional to the node support accessed by aLRT estimates. A) Full phylogenetic tree and B) Zoo med one clade highlighted in the outermost bars from part A. Online data about Penelope phylogeny is available on: https://itol.embl.de/tree/20013326103993151014469

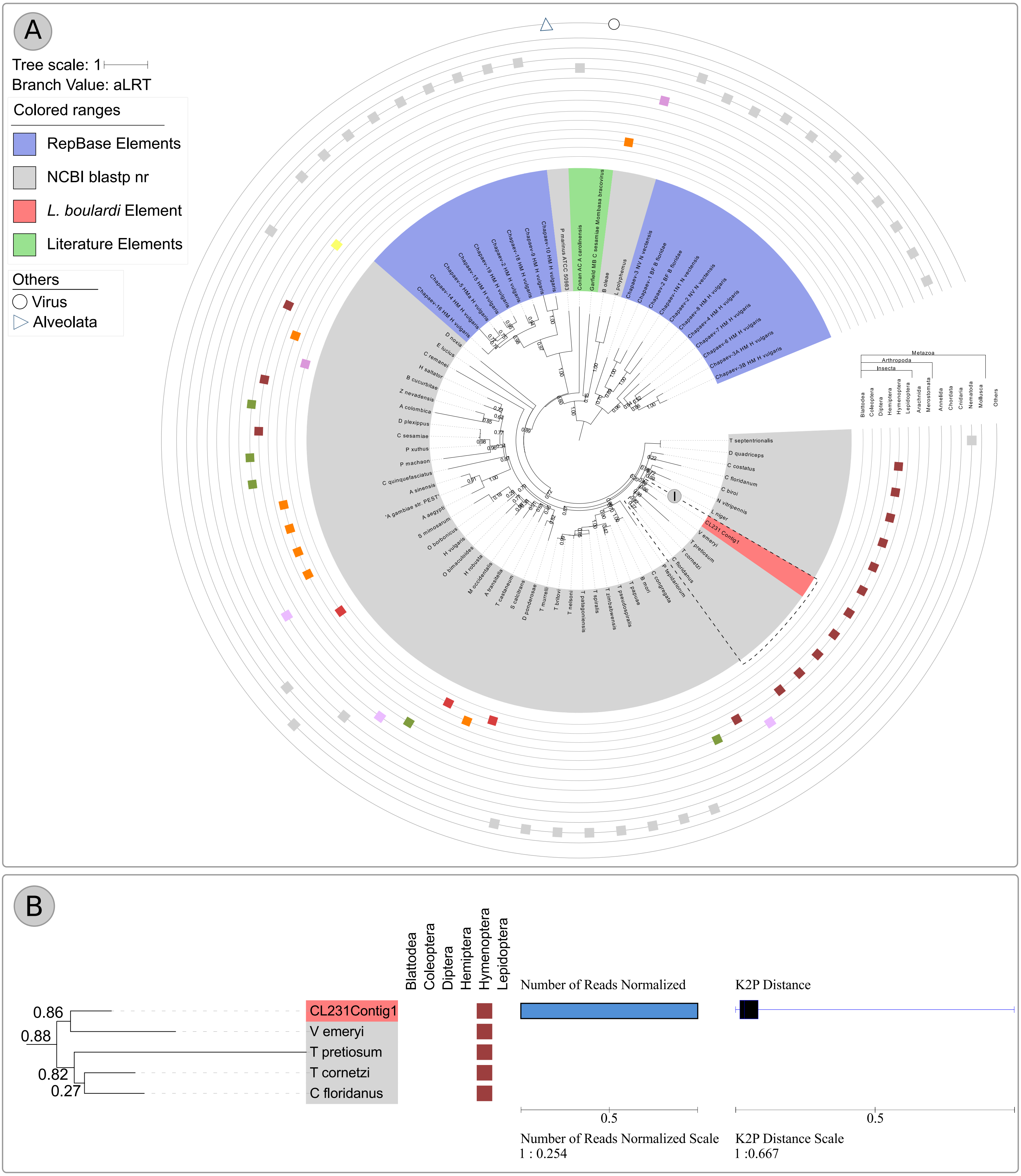

Figure 4S - Maximum likelihood of Chapaev superfamily phylogenetic reconstruction using full protein sequences from Repbase, literature and NCBI search along with Chapaev sequence from *Leptopilina boulardi*. Tip colors represent sequences recovered from RepBase - blue, from the literature - green and NCBI database - black. Triangles over nodes are proportional to the node support accessed by aLRT estimates. A) Full phylogenetic tree and B) Zoomed one clade highlighted in the outermost bars from part A. Online data about Chapaev phylogeny is available on: https://itol.embl.de/tree/2001332610128841509543832

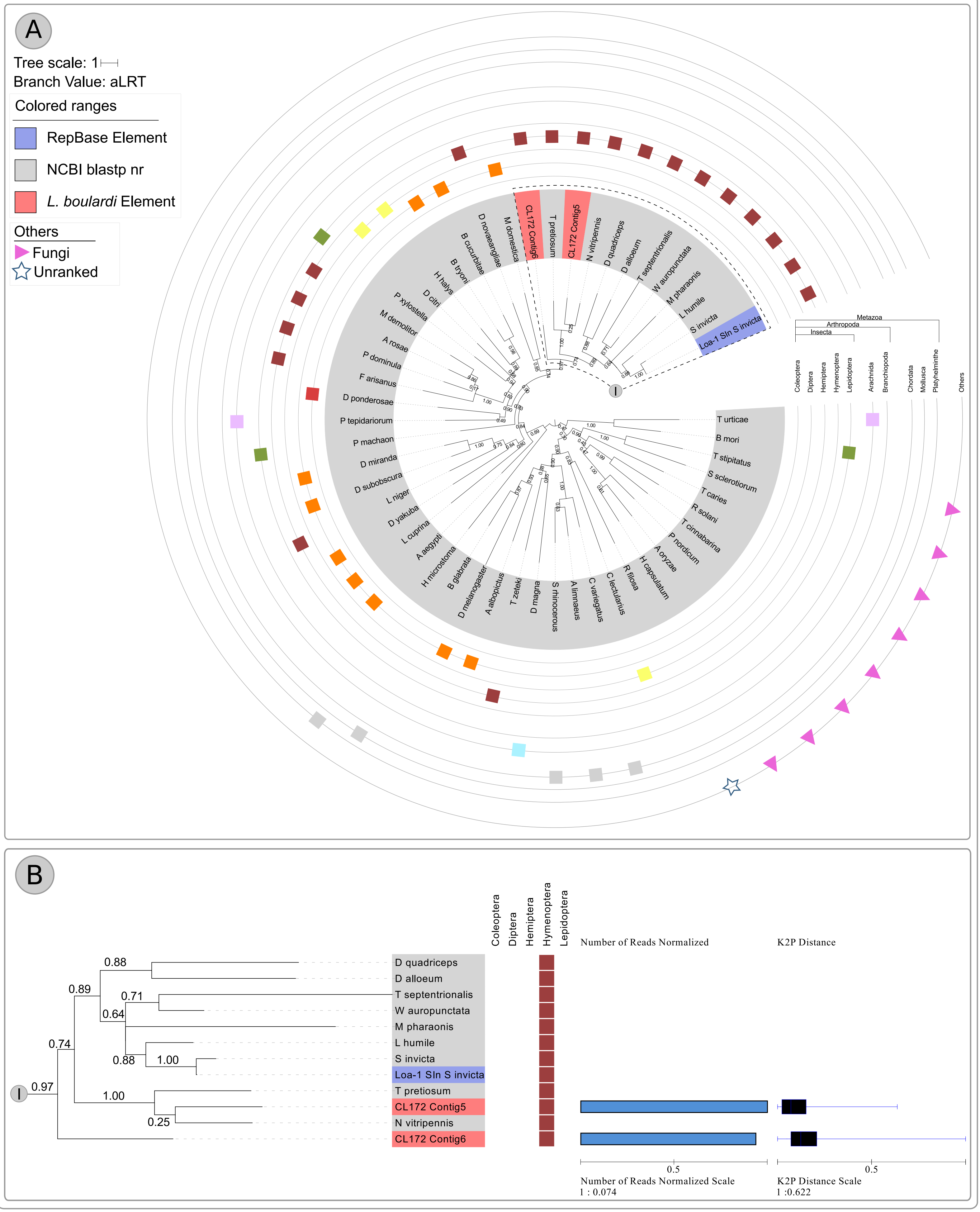

Figure 5S - Maximum likelihood of Loa superfamily phylogenetic reconstruction using full protein sequences from Repbase and NCBI search along with Loa sequence from *Leptopilina boulardi*. Tip colors represent sequences recovered from RepBase - blue, and NCBI database - black. Triangles over nodes are proportional to the node support accessed by aLRT estimates. A) Full phylogenetic tree and B) Zoomed one clade highlighted in the outer-most bars from part A. Online data about Loa phylogeny is available on: https://itol.embl.de/tree/2001321431256491510934880

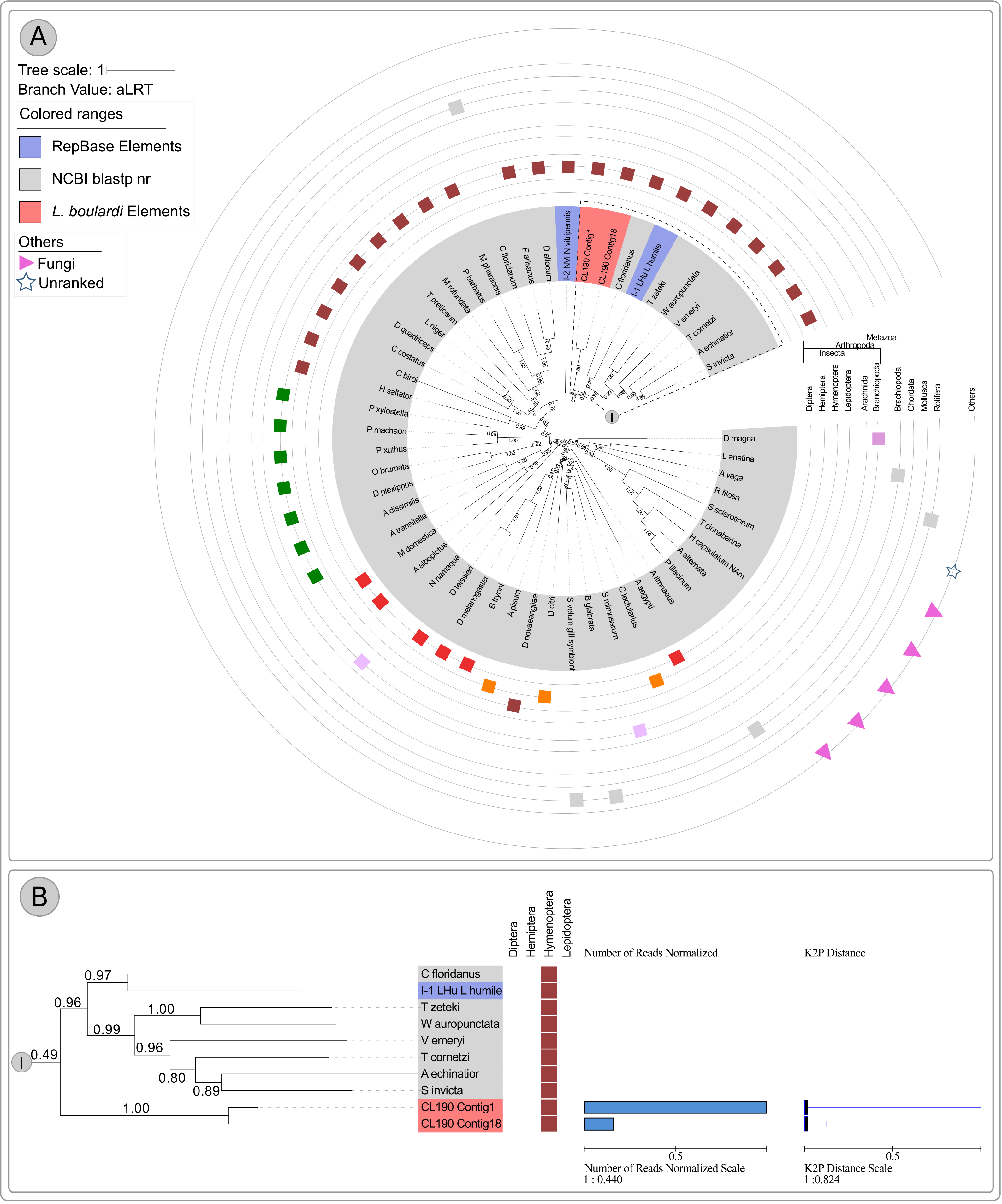

Figure 6S - Maximum likelihood of I superfamily phylogenetic reconstruction using full protein sequences from Repbase and NCBI search along with I sequence from *Leptopilina boulardi*. Tip colors represent sequences recovered from RepBase - blue, and NCBI database - black. Triangles over nodes are proportional to the node support accessed by aLRT estimates. A) Full phylogenetic tree and B) Zoomed one clade highlighted in the outermost bars from part A. Online data about I phylogeny is available on: https://itol.embl.de/tree/1893540191791915117212601

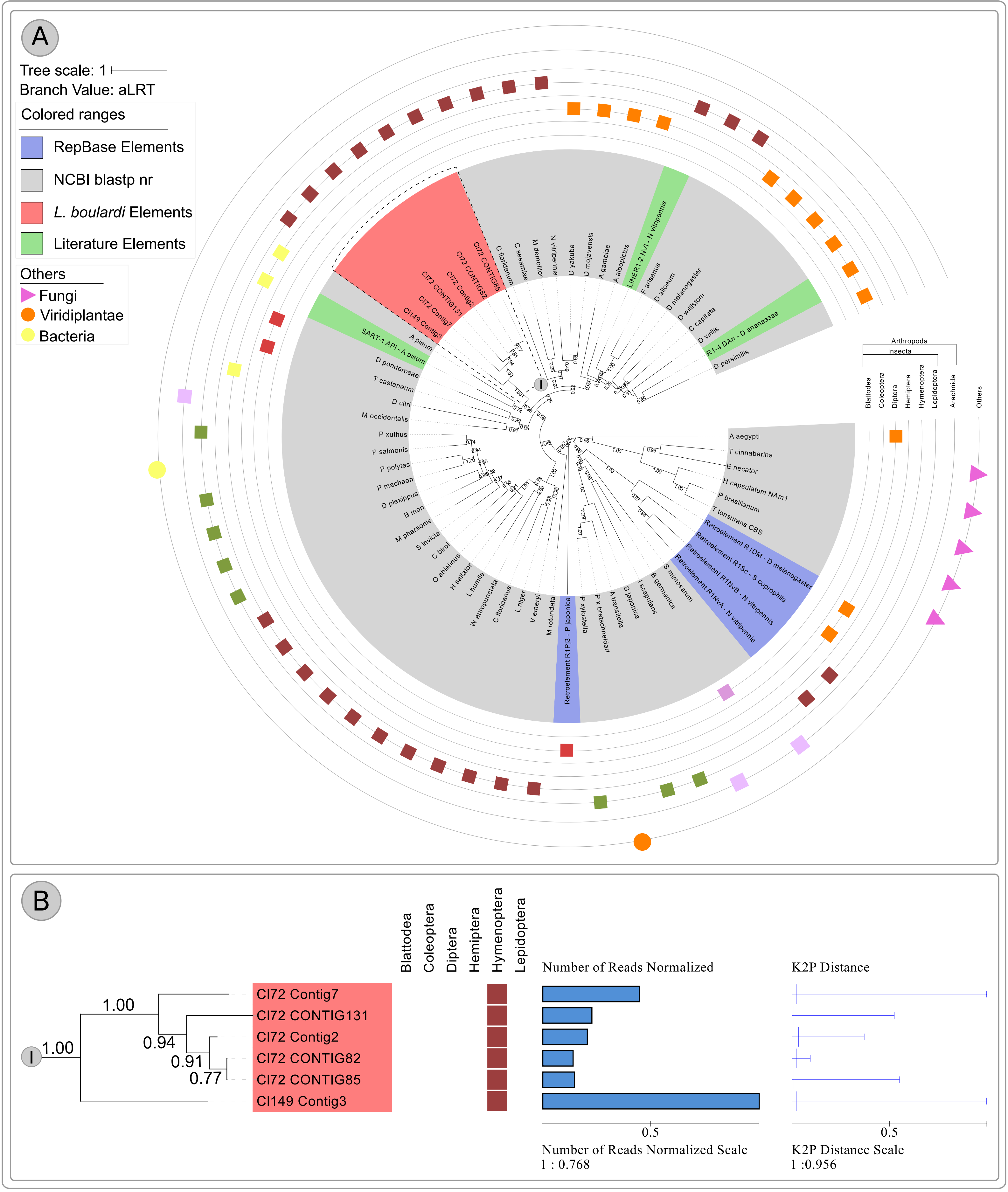

Figure 7S - Maximum likelihood of R1 superfamily phylogenetic reconstruction using full protein sequences from Repbase and NCBI search along with R1 sequence from *Leptopilina boulardi*. Tip colors represent sequences recovered from RepBase - blue, Literature - green and NCBI database - black. Triangles over nodes are proportional to the node support accessed by aLRT estimates. A) Full phylogenetic tree and B) Zoomed one clade highlighted in the outermost bars from part A. Online data about R1 phylogeny is available on: https://itol.embl.de/tree/1893540193101115117289355t
