# Supplementary material for: Drosophila parasitoid wasps bears a distinct DNA transposon profile
Source: Mob DNA. 2018 Jul 7;9:23. doi: 10.1186/s13100-018-0127-2 (PMC6035795; doi:10.1186/s13100-018-0127-2)
Supplement: Supplementary file 9 — Copia superfamily tree reconstructed by maximum likelihood. Numbers on branches denote the aLRT branch support. (PDF 641 kb) [file 13100_2018_127_MOESM9_ESM.pdf]

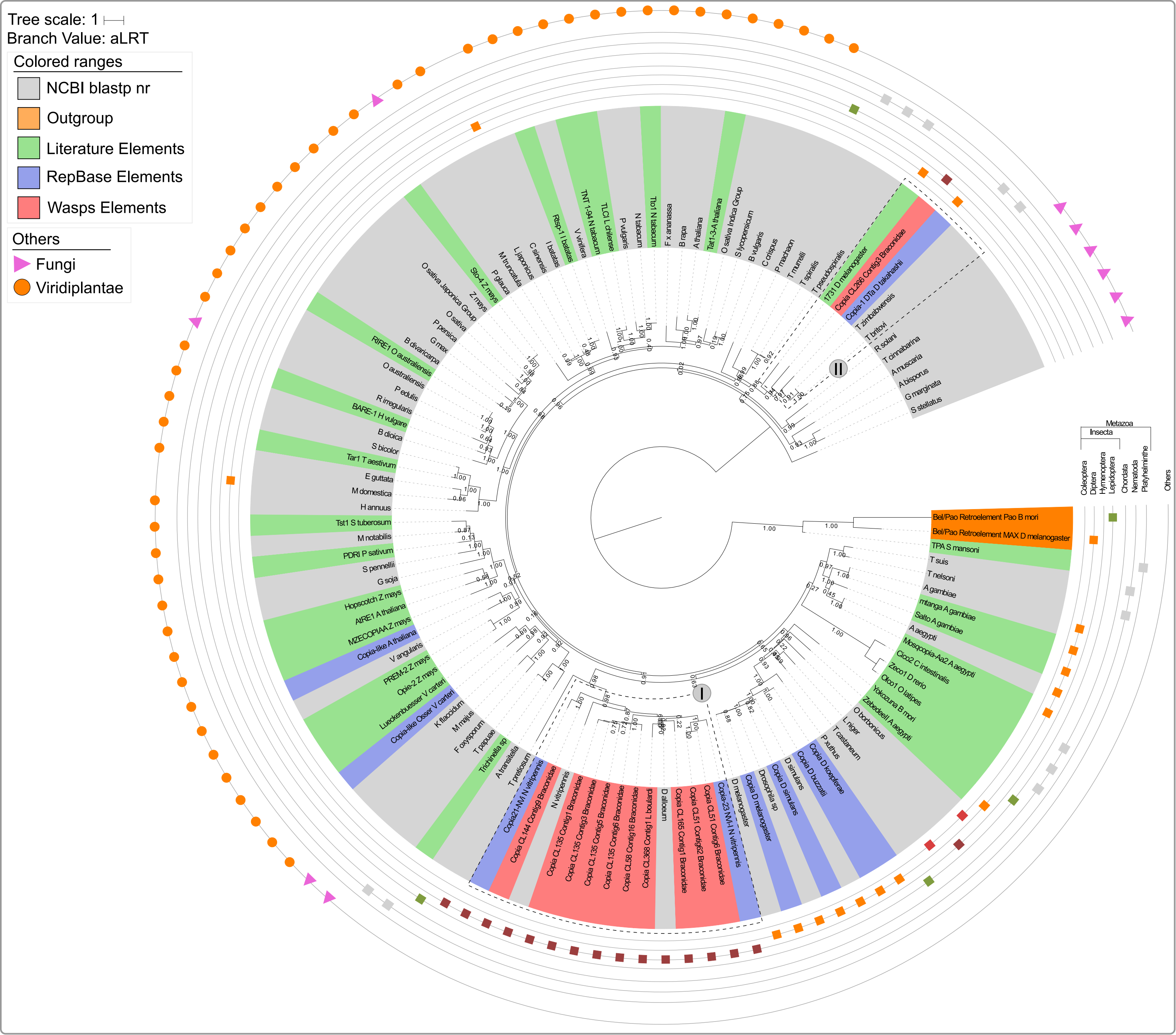
Tree scale: 1
Branch Value: aLRT
Colored ranges
NCBI blastp nr
Outgroup
Literature Elements
RepBase Elements
Wasps Elements
Others
Fungi
Viridiplantae
Metazoa
Insecta
Coleoptera
Diptera
Hymenoptera
Lepidoptera
Chordata
Nematoda
Platyhelminthe
Others
I
II
Bel/Pao Retroelement Pao B mori
Bel/Pao Retroelement MAX D melanogaster
TPA S mansoni
T suis
T nelsoni
A gambiae
mtanga A gambiae
Salto A gambiae
A aegypti
Mosqcopia-Aa2 A aegypti
Cico2 C intestinalis
Zeco1 D rerio
Olco1 O latipes
Yokozuna B mori
Zebedeell A aegypti
O borbonicus
L niger
T castaneum
P xuthus
Copia D koepferae
Copia D buzzatii
Copia D simulans
D simulans
Drosophila sp
Copia D melanogaster
D melanogaster
Copia-23 NV-I N vitripennis
Copia CL51 Contig6 Braconidae
Copia CL51 Contig62 Braconidae
Copia CL165 Contig1 Braconidae
D alloeum
Copia CL368 Contig1 L boulardi
Copia CL58 Contig16 Braconidae
Copia CL135 Contig6 Braconidae
Copia CL135 Contig5 Braconidae
Copia CL135 Contig3 Braconidae
Copia CL135 Contig1 Braconidae
N vitripennis
Copia CL144 Contig9 Braconidae
Copia21-NVI N vitripennis
T pretiosum
A transitella
Trichinella sp
T papuae
F oxysporum
M majus
K flaccidum
Copia-like Osser V carteri
Lueckenbuesser V carteri
Opie-2 Z mays
PREM-2 Z mays
V angularis
Copia-like A thaliana
MZECOPIAA Z mays
AtRE1 A thaliana
Hopscotch Z mays
G soja
S pennellii
PDRI P sativum
M notabilis
Tst1 S tuberosum
H annuus
M domestica
E guttata
Tar1 T aestivum
S bicolor
B dioica
BARE-1 H vulgare
R irregularis
P edulis
O australiensis
RIRE1 O australiensis
B divaricarpa
G max
P persica
O sativa
O sativa Japonica Group
Z mays
Sto-4 Z mays
P glauca
M truncatula
L japonicus
C sinensis
I batatas
Rtsp-1 I batatas
V vinifera
TNT 1-94 N tabacum
TLC1 L chilense
P vulgaris
N tabacum
Tto1 N tabacum
F x ananassa
B rapa
A thaliana
Tat1-3-A thaliana
O sativa Indica Group
S lycopersicum
B vulgaris
C crispus
P machaon
T murrelli
T spiralis
T pseudospiralis
1731 D melanogaster
Copia CL266 Contig3 Braconidae
Copia-1 DTa D takahashii
T zimbabwensis
T britovi
R solani
T cinnabarina
A muscaria
A bisporus
G marginata
S stellatus
