# Supplementary material for: Drosophila parasitoid wasps bears a distinct DNA transposon profile
Source: Mob DNA. 2018 Jul 7;9:23. doi: 10.1186/s13100-018-0127-2 (PMC6035795; doi:10.1186/s13100-018-0127-2)
Supplement: Supplementary file 8 — Helitron phylogenetic tree with complete coding regions of RepBase elements. (PDF 59 kb) [file 13100_2018_127_MOESM8_ESM.pdf]

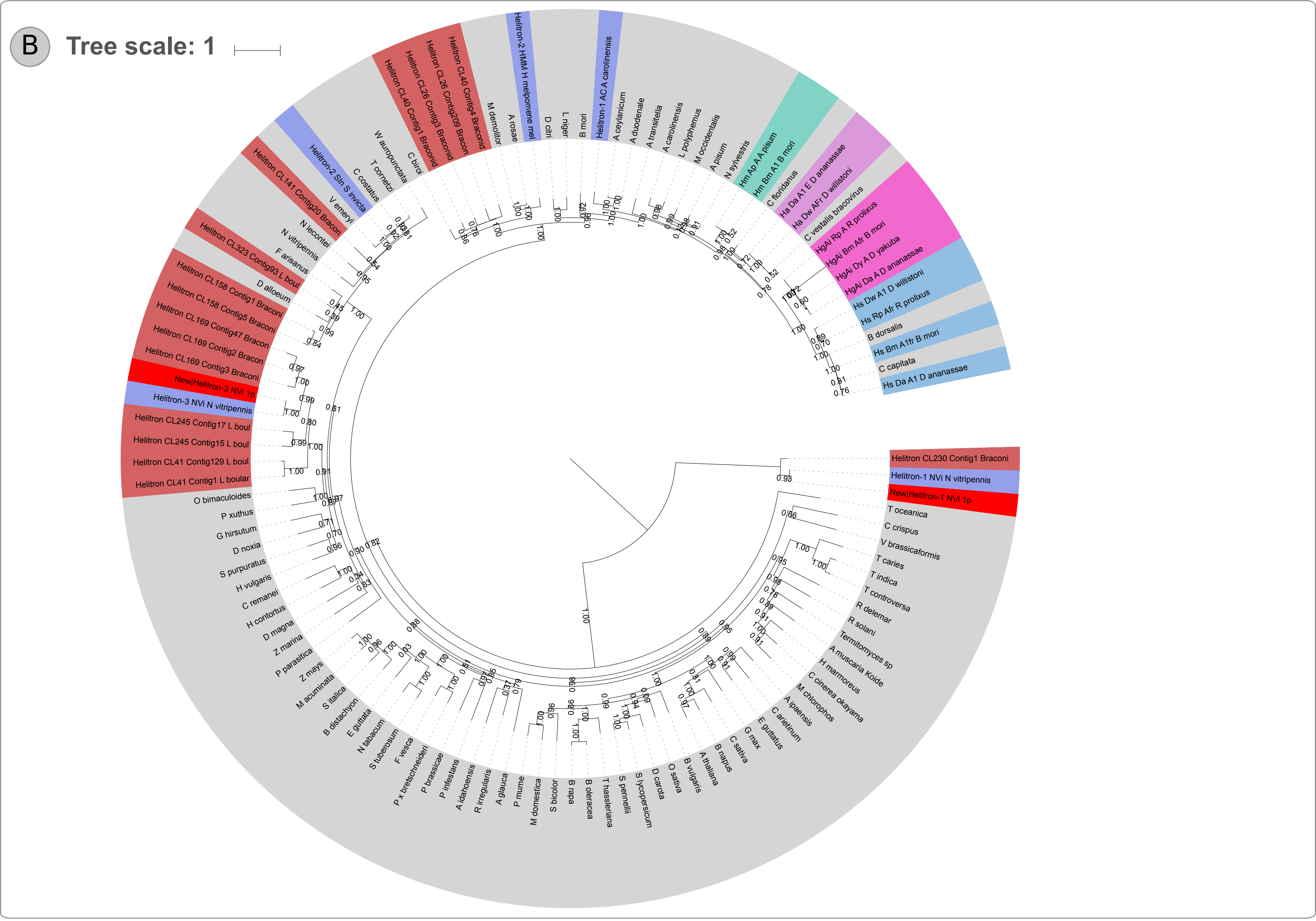

Figure 8S - Helitron phylogenetic tree with complete coding regions of RepBase elements.
